# Designing Work with People Living with Dementia: Reflecting on a Decade of Research

**DOI:** 10.3390/ijerph182211742

**Published:** 2021-11-09

**Authors:** Paul A. Rodgers

**Affiliations:** Department of Design, Manufacturing and Engineering Management, University of Strathclyde, 75 Montrose St., Glasgow G1 1XJ, UK; paul.rodgers@strath.ac.uk; Tel.: +44-(0)141-548-2091

**Keywords:** design, co-design, dementia, disruption, care

## Abstract

The United Nations Universal Declaration of Human Rights is widely acknowledged as a landmark document in the history of human rights. Drafted by representatives from all over the world, the declaration was proclaimed by the United Nations General Assembly in Paris on 10 December 1948 (General Assembly resolution 217 A) as a common standard for all peoples and all nations. The declaration sets out a series of articles that articulate a number of fundamental human rights to be universally protected. Article 23 of the declaration relates to the right to work and states that people have a human right to work, or engage in productive employment, and may not be prevented from doing so. The right to work is enshrined in international human rights law through its inclusion in the International Covenant on Economic, Social and Cultural Rights, where the right to work emphasizes economic, social and cultural development. This paper presents ongoing research that highlights how a disruptive co-design approach contributes to upholding UN Article 23 through the creation of a series of innovative working practices developed with people living with dementia. The research, undertaken in collaboration with several voluntary and third sector organizations in the UK, looks to break the cycle of prevailing opinions, traditional mindsets, and ways-of-doing that tend to remain uncontested in the health and social care of people living with dementia. As a result, this research has produced a series of innovative work opportunities for people living with dementia and their formal and informal carers that change the perception of dementia by showing that people living with dementia are capable of designing and making desirable products and offering much to UK society after diagnosis. In this ongoing research, the right to continue to work for people living with dementia post-diagnosis in creative and innovative ways has clearly helped to reconnect them to other people, helped build their self-esteem, identity and dignity and helped keep the person with dementia connected to their community, thus delaying the need for crisis interventions. This paper reports on a series of future work initiatives for people living with dementia where we have used design as a disruptive force for good to ensure that anyone diagnosed with dementia can exercise their right to work and engage in productive and rewarding employment.

## 1. The Nature of Dementia

Dementia is a syndrome where there is, usually, a progressive deterioration in the individual’s cognitive function beyond what might be expected from normal ageing. Dementia can have an effect on a person’s memory, their thinking, orientation, comprehension, learning capacity, language, and judgement. Dementia results from a variety of diseases and injuries that primarily or secondarily effect the brain such as Alzheimer’s disease, which is the most common form of dementia and may contribute to approximately 60% to 70% of cases. Other major forms include vascular dementia, dementia with Lewy bodies (abnormal aggregates of protein that develop inside nerve cells), and a group of diseases that contribute to frontotemporal dementia (degeneration of the frontal lobe of the brain). Dementia can be overwhelming, not only for the people who have it, but also for their carers and their families. There is often a lack of awareness and understanding of dementia, resulting in stigmatization and barriers to diagnosis and care. The impact of dementia on carers, family and society at large can be physical, psychological, social and economic. 

Dementia affects a person’s memory, their cognitive abilities and their behaviour, which ultimately inhibits their ability to undertake day-to-day activities. It is a major cause of disability and dependency among older adults all over the world. Worldwide, there are around 50 million people living with dementia, and there are nearly 10 million new cases every year. Nearly 60% live in low- and middle-income countries. The total number of people with dementia is projected to reach 82 million in 2030 and 152 million in 2050 (Alzheimer’s Disease International, 2020). The majority of this increase is attributable to the rising numbers of people with dementia living in low- and middle-income countries. Consequently, dementia has significant social and economic implications in terms of the costs of specialist health and social care. In 2015, the total global societal cost of dementia was estimated to be over $800 billion, equivalent to 1.1% of global gross domestic product (GDP) [1]. The estimated total global societal cost of dementia was revised in 2019 to US$1.3 trillion, and these costs are expected to surpass US$2.8 trillion by 2030 as both the number of people living with dementia and care costs increase [2]. 

The impact of dementia on people living with dementia, their family members and their carers can be even more overwhelming. The physical, emotional and financial pressures can be a cause of enormous stress and worry to the family members of people living with dementia and their carers as they locate, access and meet the significant costs associated with the health, social, financial and legal systems needed. Dementia affects each person in a different way [3]. The signs and symptoms linked to dementia can be understood in three stages:

- The early stages of dementia are often missed or overlooked, because the onset is gradual. Common symptoms include forgetfulness, losing track of time, and becoming lost in familiar places;
- The middle stages include clearer and more significant signs and symptoms such as the individual becoming forgetful of recent events and people’s names, having increasing difficulty with communication, needing help with personal care, experiencing behaviour changes, and repeated questioning;
- The later stages involve near total dependence and inactivity. Here, physical signs and symptoms become more obvious and include becoming unaware of time and place, having difficulty recognizing friends and family members, having an increasing need for personal care, and experiencing behaviour changes that may escalate and include aggression.

Although numerous new treatments are being investigated in various stages of clinical trials across the world, there is no treatment currently available to cure dementia or to alter its progressive course. There is, however, much that can be done to support and improve the lives of people living with dementia, their family members, and their formal and informal carers. People with dementia are frequently denied their basic human rights [3], so in response the World Health Assembly endorsed the World Health Organization’s (WHO) global action plan on the public health response to dementia 2017 to 2025 that provides a comprehensive blueprint for action including policies, programmes, interventions and actions that are sensitive to the needs, expectations and human rights of people living with dementia, consistent with the Convention on the Rights of Persons with Disabilities and other international and regional human rights instruments. 

The World Health Organization’s global action plan for people living with dementia comprises a series of cross-cutting principles that aim to empower and engage people living with dementia and their carers in policy-making, planning, service provision, and research. Moreover, the World Health Organization plan highlights the importance in designing and developing strategies and interventions for dementia care that are person-centred, sustainable and affordable, and take public health principles and cultural aspects into account. The following sections of this paper will describe a series of carefully designed interventions (i.e., products, services, systems, strategies), developed through a suite of projects, aimed at supporting people living with dementia and their carers that aim to improve their lives. 

## 2. Disrupting Health and Social Care via Design Acts

The overarching aim of this research is to develop disruptive design interventions (e.g., products, systems, services, strategies) for breaking the cycle of established opinions, strategies, mindsets, and ways-of-doing, that tend to remain unchallenged in the health and social care of people living with dementia in the UK. The long-term goal of this research, however, is to uphold UN Article 23 that states that people have a human right to work, or engage in productive employment, and may not be prevented from doing so. Through the co-design and development of a range of products, methods, strategies and tools, we aim to provide people living with dementia supported by their formal and informal carers with innovative and creative work opportunities that shift widely-held opinions of dementia by showing that people living with dementia are capable of designing and making desirable products and offering much to society after diagnosis. Moreover, “good” employment will address adverse effects of lack of stimulation amongst people living with dementia by upholding their right to continue to work in creative and innovative ways that will help keep them connected to other people in their community, help build their self-esteem and retain their dignity, thus delaying inputs from more formal support and crisis interventions [4]. This paper reports on a series of future work initiatives for people living with dementia, developed over more than 10 years, where the author has used design as a disruptive force for good to ensure that anyone diagnosed with dementia can exercise their right to work and engage in productive and rewarding employment.

## 3. The Disruptive Design Approach

Against a backdrop of the global financial crises of 2007 and the United Kingdom government’s austerity programme that started in late 2008, the Design Disruption Group was formed by the author and three of his colleagues at Northumbria University, School of Design—Andy Tennant, Giovanni Innella and Freddie Yauner. At this time, David Cameron’s Conservative government initiated a range of programmes that aimed to end years of what they characterised as excessive government spending [5]. The deficit reduction programme consisted of sustained reductions in public spending aimed at reducing the government budget deficit and the role of the welfare state in the United Kingdom at this time. The austerity measures resulted in substantial reductions in public spending, principally through budgetary cuts on departments and services, which significantly affected local government funding and support—particularly in health and social care contexts. 

Given the significant challenges faced by many voluntary and third-sector organizations that started around late 2007 to early 2008 in the UK, the Design Disruption Group set out to support care organizations, informal carers and care workers in the North East of England through a series of disruptive design workshops aimed at breaking the cycle of well-formed opinions, mindsets, and ways of doing things that tend to remain unchallenged in health and social care contexts. Around this time, in the UK, almost 6 million people were classed as unpaid informal carers for an ill, frail or disabled family member or friend who are unable to manage to live independently or whose health or wellbeing would deteriorate without their help. In the UK, informal carers save the UK Government £132 billion a year and nearly 7 million households contain an unpaid informal carer, which represents huge social care and NHS cost savings [6]. Although the role and experience of informal carers is unique to their situation, and caring can be a rich source of satisfaction, it is also known that their health suffers and that they have an increased rate of physical health problems.

The disruptive design approach we developed and use encourages the development of richer, more varied solutions to everyday issues by emphasizing fun [7], “safe failure” [8], and doing things in ways that those working with people with dementia would not normally do. Safe failure involves small-scale experiments that allow a researcher to approach an issue from a variety of angles in small and “safe to fail” ways to allow emerging possibilities to become observable. They are not just random stabs in the dark. The emphasis is not entirely on ensuring success or avoiding failure but rather on building one’s knowledge of the issues at hand and the appropriateness of design ideas by allowing those ideas that are not useful to fail in small, managed and tolerable ways. The ideas that do result in observable benefits meanwhile can be enhanced and amplified. 

The dictionary definition of the term “disruption”, from the Latin disrupt literally means “broken apart”, to interrupt (e.g., an event, activity, or process) by causing a disturbance or a problem and/or drastically alter or destroy the structure of something. For example: “*…the rail strike has disrupted scores of passengers commuting journeys and freight services today.*” Although the term disruption tends to be synonymous with protest, dissent, activism, violence, and often is typically linked with negative connotations, we view our disruptive design interventions as a positive act and as something that can help enact change for the better. The role of the Design Disruption Group is as an active conduit between policy makers, regulatory bodies, regional controlling groups and manufacturers and local people, citizens, and excluded groups whose voice is perhaps overlooked. The Design Disruption Group see their role as creative interventionists—designing disruptive interventions that provoke discussions between the policy makers and the citizens—by creating products and services that disrupt, stimulate debate, and provoke positive change for the people, with the people and by the people.

The work described here, which has been developed over many years, espouses an innovative, interventionist methodology that is based on theories originating from research in economics [9], business [10], and design [11]. The disruptive design approach advocates direct design action that privileges working directly with stakeholders (e.g., clients, collaborators, end-users), doing and making things in order to learn, valuing failure, and iterating rapidly involves three key stages:

(1) Observe, Observe, Observe—ends our over-reliance on conventional sources of information such as Internet searches and downloading “known” knowledge and challenges one to totally immerse oneself in the “real” places (e.g., homes, shops, bus stops, pubs) that matter most to the issue(s) people are dealing with;
(2) Withdraw and Reflect—asks one to share and reflect on the situations and issues one has observed and learn from everything and everybody. The key question here is how can the designer/researcher become a part of the future world vision as opposed to holding on to and privileging past and present worldviews;
(3) Act, Make and Change in an Instant—requires one to explore the future by acting; develop interventions that help us explore the future by doing, generating feedback from all the key stakeholders that allow novel ideas to evolve.

Over the next six sections of the paper, a series of disruptive design workshops with health and social care stakeholders (i.e., people living with dementia, formal and informal carers, health professionals) will be described. Table 1 summarizes the details of each workshop. 

All of the work conducted by the author described in this paper applied for and received formal University Research Ethics Committee approval, always with additional formal approval from the collaborating partner organizations. 

Moreover, a set of guiding principles have been applied over the course of this work in relation to the co-design processes that are made clear to all participants at the outset. These are highlighted at the start of all co-design projects and appear in the consent process and in the facilitator’s introduction to workshops. These values have been applied consistently across all workshops underpinning the disruptive design approach. These values stress that (design) researchers should always ask people living with dementia how they wish to be involved in the co-design projects and ensure that they are involved in setting research priorities. In addition, (design) researchers must ask people living with dementia what positive outcomes of the research project might look like for them and they must ensure that everyone taking part in the research is physically and emotionally safe at all times, ensuring that they use language that is supportive of people living with dementia whilst avoiding language that may offend. Design researchers need to be “dementia aware”—they should be compassionate, tolerant, understanding, and respectful at all times whilst co-designing with people living with dementia.

## 4. Disrupting Carers

**Figure 1:**
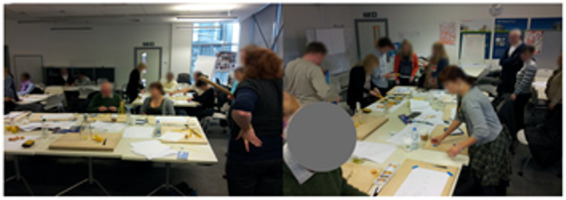
Early disruptive design workshops with carers.

The workshops provided a safe space for care organizations, such as Newcastle Carers, to develop richer, more varied solutions and possibilities to the myriad issues they faced on a daily basis. The intention behind these early workshops was for participants (in this case, carers) to have fun, relax, and do things in ways that they would not normally do and provide opportunities for the participants to try new things out in a relaxed, stress-free environment (Figure 1). 

Every disruptive design workshop is tailor-made for the organization we are collaborating with. Past workshops have included a range of creative activities, such as drawing from memory, sketching everyday observations, 2D/3D modelling, and problem-solving challenges. The goals of the disruptive design workshops are to introduce the carers to new methods of seeing and doing their day-to-day tasks, to establish trust, decrease anxiety, to promote imagination, and risk-free failure. Moreover, the workshops aim to encourage, support and positively reinforce informal carers in respect of engagement and/or re-engagement with their creative capacities and depart from their pre-conceptions and orthodoxies about how things are and/or should be to how things might be.

Caring for someone can be an exceptionally demanding role. Informal carers (usually a family member) can feel forced to give up regular employment in order to provide care and support for a loved one. Typically, in the absence of a regular income, carers are forced to rely on limited savings and meagre benefits such as Carers Allowance (UK) that amounts to £67.25 per week for a minimum of 35 h work—the equivalent of £1.92 per hour [12]. The emotional impact of caring, however, can be even more debilitating. Informal carers speak of social isolation and marginalization, unable to pursue activities that once gave their lives meaning. They are helpless to intervene as the person they care for—who may be a cornerstone of the carer’s own self-identity—undergoes significant and irreversible changes mentally and physically. This usually leads to a “biographical disruption” [13] as people experiencing dementia, as well as their carers, struggle to make sense of their new circumstances and unexpected limitations forced upon them. Dementia subverts how we understand our lives as linear paths of experience and accrual, shrouding and then erasing elements of a person. This contributes to an ongoing sense of grief without closure, or “ambiguous loss” [14]. The cognitive atrophy of dementia affects carers as much as those diagnosed with the condition. It would be misleading, however, to characterize informal care solely in negative terms. Some carers report positive feelings including a sense of greater closeness to the person they care for and enhanced self-esteem [15]. 

Given the extremely challenging nature of informal caring in the UK, we worked closely with Newcastle Carers in their ambition for carers in Newcastle and across the North East of England to:

(1) Be recognized and valued;
(2) Have access to appropriate support, information and advice;
(3) Have choice and control.

In so doing, Newcastle Carers wished to design their service provision that was:

Professional—provided a high quality, confidential service that is well governed and managed;

Equal—services and support will be accessible to all;

Respected—treating all carers as individuals;

Innovative—creating and developing new ways of working and delivering services to support the needs of carers and stakeholders;

Adaptable—flexible and responsive;

Collaborative—sharing knowledge and resources, and building and maintaining meaningful relationships with other organizations;

Empowering—enabling carers to have choice and control in identifying and achieving their own goals;

Person-centred—delivering a service based on the needs of carers and the people they look after;

User-led—carers are at the heart of the organization through governance, planning and consultation.

Our work with carers resulted in a number of positive outcomes. Initially, the carers expressed some reservations about the nature and scope of the workshops. Several of them stated that they were “…excited but nervous because they didn’t know what to expect…” whilst others stated that “…they wished they were more creative…” and “…that they didn’t like not being in control…”. This is exactly the point of the workshops; they are intended to disrupt and take the workshop participants out of their comfort zone. In general, however, the feedback from the carers has reinforced the positive results from the workshops. The carers have stated that the workshops were novel, informative and a lot of fun for them. They also said that the workshops were a great opportunity for them to bond more with their colleagues and for them to get away from the demands of their very stressful jobs. For many of them, the main thing that they learnt from the workshop was that it is good to take risks sometimes, think differently, and that failing can be a highly positive experience. 

## 5. Disrupting Dementia

**Figure 2:**
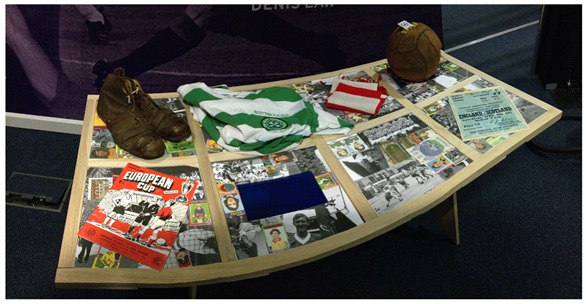
Interactive Scottish football table.

For the next five to six years, the Design Disruption Group established a number of projects and worked with a wide range of collaborators including Gateshead Carers Association, Newcastle Carers, North East Together (Regional Mental Health Network), Newcastle YMCA, Scottish Mental Health Research Network, Alzheimer Scotland, Making Space, Mental Health Foundation, Royal British Legion, and Ryder Architects. Around this time, the author instigated several projects with collaborators in the dementia sector including dementia researchers, such as Professor Debbie Tolson and Dr. Andy Lowndes at Glasgow Caledonian University and Dr Mariesha Jaffray at Aberdeen University. One of the key projects at this time was the design and development of an interactive Scottish Football table exhibition piece (Figure 2), developed in collaboration with the Scottish Football Museum, for the project “Memories FC: The Football Reminiscence Knowledge Exchange Partnership” held at the Headquarters of Scottish Football—Hampden Park in November 2012.

The interactive Scottish football table uses the power of football to promote reminiscence. Physical museum artefacts, such as football boots, football, scarf, shirt, programme and ticket shown in Figure 2 are tagged with an RFID (Radio-frequency identification) tag each containing specific digital content that is played back via an embedded video monitor in the interactive table surface. The interactive Scottish football table was designed predominately for men who had lost some of their cognition and their ability to talk and perform; who had lost their confidence and had retracted into themselves, and tapped into the men’s intact memories (in this case around football) that was very affirming for them. This project also promoted intergenerational dementia awareness, and encouraged over 3000 museum visitors, local football clubs, fans and communities to become more involved with people living with dementia in their communities.

## 6. Co-Designing with People Living with Dementia

In November 2014, the author was awarded a prestigious Arts and Humanities Research Council (AHRC) Design Research fellowship. The key aim of this fellowship was to understand better how design thinking and action can contribute to the development of a range of enhanced products, services, and systems for people living with dementia. The fellowship entitled “Designing Innovative Interventions with People Living with Dementia” was undertaken in collaboration with Alzheimer Scotland, people living with dementia across Scotland, their formal and informal carers, the Scottish Dementia Working Group, and other relevant bodies. Around this time, there were an estimated 800,000 people living with dementia in the UK costing over £23 billion per year to the UK government. The contribution made by carers in the UK, however, is estimated to be a massive £132 billion per year or £15.1 million every hour. 

Responding to the challenges of supporting people with dementia to live well from the early stages of the illness requires innovative ways of doing things. Consequently, this fellowship focused on the key early stages of an individual’s dementia journey aiming to:

1. Help alter the perception of dementia by showing people with dementia they can offer much to UK society after diagnosis;
2. Create a series of designed interventions that will reconnect people recently diagnosed with dementia to help build their self-esteem, identity and dignity;
3. Develop interventions that will provide ongoing benefits in keeping the person with dementia connected to their community, delaying the need for formal support and avoid the need for crisis responses;
4. Explore how prevention and early intervention might enable both carers and people living with dementia to have greater choice and control in their lives;
5. Work with people recently diagnosed and their carers to participate in the creation of the designed interventions and develop and test these interventions in a number of Alzheimer Scotland’s Dementia Resource Centres and Dementia Cafes located throughout Scotland.

The author’s AHRC Design Research fellowship adopted a disruptive design interventionist approach, which celebrates jumping straight in, doing things in order to learn new things, and valuing failure. This approach was used in a series of disruptive design workshops held across Scotland with people living with dementia, their carer(s), and care support staff from Alzheimer Scotland. From the outset, this work looked to engage with both academic and public audiences in an effort to increase the accessibility of the research to the general public. This was achieved by disseminating project activities and outcomes to the public via hands-on sessions at a number of Alzheimer Scotland’s Dementia Resource Centres and through Alzheimer Scotland connections within local, reginal and national communities. The development and evaluation of the designed interventions in this work has helped to address the significant lack of evidence on outcomes and the current state of service delivery for people living with dementia and their carer(s) in the UK. In short, this work has helped improve the evidence base of design research in dementia care contexts.

Being embedded within Alzheimer Scotland for over one year provided the author with access to a wide range of expert practitioners and specialist staff. This also provided direct access to working directly with people living with dementia, which allowed the author to gain substantial “hands-on” experience in real, live contexts where designed interventions could be created, used, evaluated, and implemented with a wide range of stakeholders including people living with dementia and Alzheimer Scotland dementia support workers. This access substantially enhanced the author’s knowledge and understanding of how design interventions can play important and positive roles in the health and social care of people living with dementia as well as providing the wider design research community with first-hand knowledge and experiences of working with a leading dementia care service provider. As stated earlier, a key aim of this ongoing work is to exploit the potential of designing disruptive interventions that will change society’s perceptions of dementia by showing people living with dementia can offer much to UK society after diagnosis. Moreover, this research aims to help identify the major consequences of caring for people living with dementia and consider how prevention and early intervention through disruptive designed products, strategies, and services might enable both carers and people living with dementia to have greater choice and control in their lives. 

Often people living with dementia experience feelings of hopelessness and frustration believing that nothing can be done to help their situation [16]. Many people living with dementia think that they are inadequate in various aspects of their lives and this can lead to an individual losing their confidence and having low self-esteem. Many people living with dementia routinely perceive their status within society as being significantly reduced as a result of their diagnosis of dementia [17]. In general, people living with dementia are not considered capable of creating new products or services. They are, generally speaking, not seen as being able to contribute to a nation’s economy. Rather, they are typically seen as a consumer of care that draws on a nation’s resources. This ongoing work, however, sets out to dispel this assumption. Throughout the research presented in this paper, all of the co-designed projects been devised and undertaken from a “designing with” perspective where the user is not viewed as a “subject” but rather as an “active participant” [18]. The co-design projects in this work facilitate significant levels of collaboration to occur between people living with dementia, carers, and design researchers in the creation of inspiring new designed interventions, such as graphics, pattern making, product designs and services. The outcomes of this work have been experienced by the public through opportunities to purchase the final designs, by visiting exhibitions and by taking part in creative processes developed with and by people living with dementia. The projects also reveal what is possible as productive outputs by collaboration between people living with dementia, carers and design researchers. The key motivation behind all of the co-design work presented in this paper is to ensure that everyone involved is engaged fully and in ways that espouse empathy, dignity, and respect. As such, great care is taken to consult with people living with dementia, their family members, and care support workers about how they want to be involved throughout the projects before they commence. In particular, it is vital that the co-design projects support the person living with dementia and that they pay respect to their personhood and their right to be treated as unique individuals [19].

## 7. Disrupting Dementia Tartan

The Disrupting Dementia tartan co-design project was one of the projects undertaken during the author’s Arts and Humanities Research Council (AHRC) Design Research Fellowship. This project involved over 130 people living with dementia based in dementia resource centres across Scotland including Glasgow, Edinburgh, Dundee, Shetland, Orkney and Stornoway taking part in specially designed co-design workshops. The author travelled over 1900 miles across the length and breadth of Scotland, racking up over 80 h spent travelling on planes, trains and automobiles, and using over half a kilometre of coloured ribbon in the creation of the participants’ Disrupting Dementia tartan design prototypes. The major aim of the Disrupting Dementia tartan design project was to change widely held attitudes and perceptions of dementia by highlighting the creative abilities of people living with dementia. In particular, this project set out to show that with some careful and well-designed plans, preparation, and encouragement people living with dementia can contribute much to UK society after diagnosis. The Disrupting Dementia tartan project clearly illustrated that people living with dementia are capable of designing a new product with the potential of it being sold all over the world. Thus, this project contributes to the £461 m value of exported design products and services from the UK in 2017 [20]. 

It is vitally important in a co-design project such as this one to ensure that the designer (author) does not take an overly dominant role. At the outset of any co-design project, the lead researcher (designer) must be clear about the aims and objectives of the project and articulate clearly the rationale behind the project and what is expected of everyone involved. In other words, the project rationale should always be known and agreed upon from both sides. In projects such as this, the key aim is always to achieve something like a symbiotic collaboration—a mutually beneficial relationship between those involved. Van Klaveren [21] suggests such an ethical and transparent approach is the foundation for a truly symbiotic co-design relationship. Accordingly, in this project, each Disrupting Dementia tartan co-design workshop commenced with a short presentation of the project aims and requirements associated with the creation of the Disrupting Dementia tartan. Tartan was at the core of this project because it is an important part of Scottish culture and because all of the participants (co-designers) would recognize tartans and all have opinions on what makes for a good tartan as well as possessing personal attachments and stories of their own relating to tartan. At every stage of the project, the researcher (author), working closely with people living with dementia, Alzheimer Scotland staff and family members, took great care to ensure that the language used and tasks involved during the workshop was supportive and not offensive to people living with dementia. In addition, great care was taken to ensure that everyone taking part in the project was kept physically and emotionally safe at all times during the workshops. 

**Figure 3:**
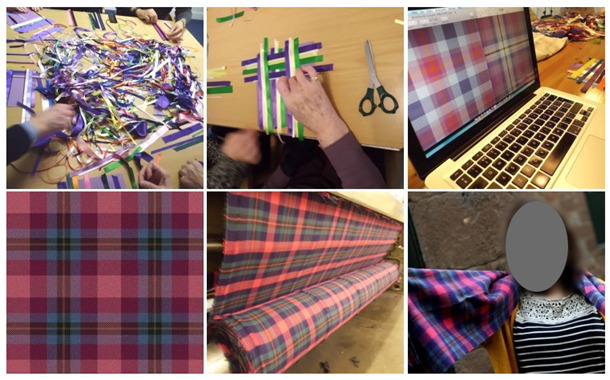
Disrupting Dementia tartan design process (**left** to **right**, **top** to **bottom**: coloured ribbon physical prototypes; initial digital prototypes; tartan manufacture; end product).

The creation of each participant’s tartan design began with a physical prototype constructed using coloured ribbon, followed by the creation of a digital version using a publicly available Internet-based tartan design tool (Figure 3). Each participant was free to determine and shape the tartan design they created during the stages of the design process.

In the example shown in Figure 3, one can see that the person living with dementia’s main colour in their design is purple (Alzheimer Scotland’s main brand colour), followed by their choice of colours. At this important stage of the co-design process, the researcher (author) adopted an empathic (not sympathetic) approach ensuring he was considerate, respectful, tolerant, and understanding of the varied abilities and skills involved (Rodgers, 2018). Here, it should be pointed out that several workshop participants held (many until recently) significant employment positions before their diagnosis of dementia, including an eye surgeon, an architect, an engineer, a baker, and many others. Thus, a respectful attitude espousing equality between all co-designers involved was vital to the success of the co-design sessions. During the highly iterative stages of the Disrupting Dementia tartan co-design project, the researcher (author) consistently had to consider “dementia time”. That is, being patient and ensuring there was enough time and space for each individual (co-designer) and how each individual keeps track of their own time. Some approaches to co-design stress the need to rethink and redefine the role of participants [21]. A vitally important point is to ensure that great care is taken to not imply power relations through the language and terms that are used in co-design projects [22]. In this project, the participants are seen as collaborating designers (authors) in the process and their input is valued as much as any other participant. Like the work of Manzini and Rizzo [23], this co-design project views the participants as active collaborative co-designers. In this project, the people living with dementia are active and equal participants; they are creative, they bring a range of valuable experiences, skills, knowledge, and capabilities and they enhance the overall nature of the project by taking part. 

## 8. 75BC

**Figure 4:**
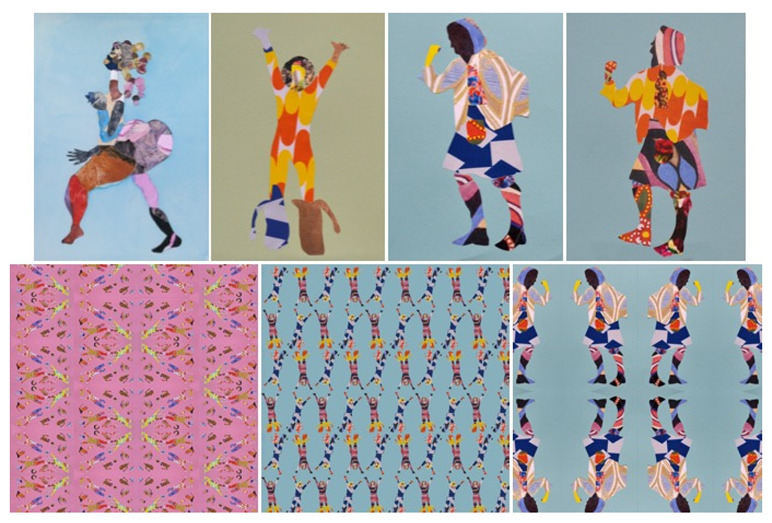
Bridgeton textile design (**left** to **right**, **top** to **bottom**: Tschabalala Self collage; Billy Connolly collages; new Bridgeton textile fabric design prototypes).

75BC is a co-design project that aims to honour the life and work of the famous Glaswegian comedian Billy Connolly. This co-design project, based in Bridgeton in the East End of Glasgow, involved a series of collaborative visits to the 75BC murals located around Glasgow and to the American artist Tschabalala Self’s exhibition at the Tramway Gallery in Glasgow. During this co-design project, five people living with dementia designed and produced a range of visual representations of Billy Connolly using visual collage techniques to create a series of textile design prototypes in the style of Tschabalala Self (Figure 4). The textile design prototypes created by people living with dementia have been inspired by a series of fabric patterns originally produced in the Bridgeton area of Glasgow (called Turkish Red) alongside patterns from the wider textile design community. Two representational forms of Billy Connolly were used here—one image shows Billy Connolly in his iconic Big Banana Boots pose from early in his career and the other image shows a more recent picture of Billy Connolly in his Dressed-to-Kilt outfit from 2011. All of the co-designers in this project composed their patterns and colour schemes in accordance with these two original representational forms. Some members of the group followed a clear strategy in their co-design project whereas others adopted a much freer expressive approach in their textile design prototypes. The key aim of the 75BC co-design project is to use the images created by the five people living with dementia to illustrate their inherent creativity and to support the wider 75BC celebrations. As the 75BC co-design project develops, the co-designers will decide themselves which of these patterns will become a new Bridgeton textile design.

## 9. Designed with Dementia

The author’s most recent work is Designed with Dementia, which aims to exploit the latent creative abilities of people living with dementia. Designed with Dementia espouses a co-design approach where people living with dementia are highly valued and treated as equals in the design and development of new products and services. Here, people living with dementia, their inputs, their judgement, decision-making, and collaborations are held in the same esteem as any other collaborator. Designed with Dementia strives to empower and include people living with dementia, along with expert dementia support workers, carers and the general public, to inform, influence and change prevailing attitudes and assumptions surrounding dementia. During the Designed with Dementia process, all participants are viewed and treated as co-designers helping to propose possibilities, select solutions, make key decisions, and generally make things happen.

**Figure 5:**
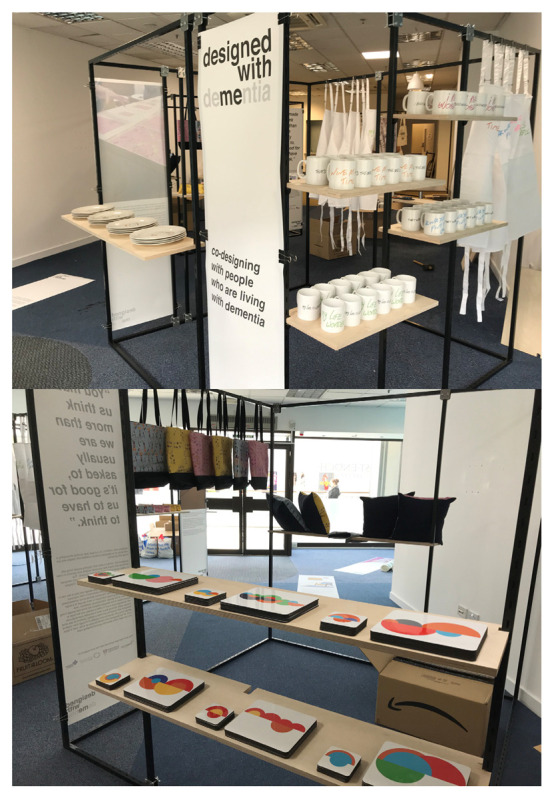
Designed with Dementia pop-up shop in Glasgow.

The main output from the Designed with Dementia project is a “pop-up” shop that has been rolled out in Lancaster and Glasgow (Figure 5). Designed with Dementia is a retail enterprise that showcases a range of products that have all been co-designed with people living with dementia. The range of Designed with Dementia products extends to ceramic ware (e.g., plates and mugs), tea towels, cooking aprons, coasters, tote bags, cushions, make-up bags, ties, and other products. The Designed with Dementia “pop-up” shop also includes a service for people living with dementia to come and explore first-hand the range of products and design and make their own tea towels and cushions in the shop. Here, people living with dementia along with their friends and family members can come and participate in the design and manufacture of a range of soft furnishing designs depicting images of famous local people and/or well-known landmarks. Alongside the designing and making facility, visitors to the “pop-up” shop will be able to view and purchase a range of products created in earlier Designed with Dementia events. The Designed with Dementia “pop-up” shops provide an accessible and inclusive form of public exhibition that helps to highlight the creative potential of people living with dementia and how this form of work with people living with dementia can be implemented across local, regional and national contexts and extended to the design and manufacture of commercial products [24].

## 10. Reflections

This paper looks back on more than a decade of the author’s work with people living with dementia. As a design researcher, the author routinely adopted a combination of “reflection-in-action” [25] and “reflection-on-action” [26] in these projects. That is, thinking and reflecting whilst doing (designing). For example, whilst in the act of co-designing with people living with dementia occasionally changes would need to be made during the co-design sessions, such as swapping modelling materials, re-ordering the stages of a particular task, or eliminating some activities altogether. In terms of reflection-on-action, the author continually assessed the impact and effects of the co-designing projects against predetermined aims and objectives [26] agreed with the participants. Here, the author would conduct both analytical reflection (i.e., reflecting on how things were done) and evaluative reflection [27]. Evaluative reflection involved making decisions and judgements in relation to criteria that were set at the outset of the project/activity. Often in evaluative reflection processes, the judgement and decision-making leads to new decisions and consequences (e.g., deciding to change one material with a new one and deciding to remove particular design process stages from the overall co-design project). 

One area of this work that will be of interest to those who may wish to apply this methodological approach of disruptive design relates to ethical considerations, particularly those participants with dementia and their carers. Ethics in design research, particularly in projects with people living with dementia, is vitally important. At the start of any co-design project, you should clarify your research intentions with your participants. In all of the co-design projects presented in this paper, the people living with dementia are viewed as collaborative designers who have equal decision-making powers and ownership of the designed outcomes. In collaborative design projects such as the ones described in this paper, it is vital that everyone is treated equally, with courtesy at all times, and a consistent, non-judgmental, relaxed and harmonious environment is maintained throughout the design process [28,29,30,31]. 

## 11. Conclusions

This paper posits that people living with dementia have the right to live as full citizens and have full participation and inclusion in all aspects of life. This includes the right to work, enshrined in the United Nations Universal Declaration of Human Rights (Article 23 of the declaration, which states that people have a human right to work, or engage in productive employment, and may not be prevented from doing so). In this ongoing research, the right to continue to work for people living with dementia post-diagnosis in creative and innovative ways has clearly helped to reconnect them to other people, supported the development of their self-confidence, character and dignity and helped keep the person living with dementia connected to their friends and family members, thus suspending the need for more formal health and social care support and the need for crisis interventions. The paper has presented a series of future work initiatives, developed over many years, for people living with dementia where design has been used as a disruptive force for good to ensure that anyone diagnosed with dementia can exercise their right to work and engage in productive, creative, and rewarding employment.

The paper has highlighted a series of ground-breaking work undertaken by the author in collaboration with many individuals including innovative co-design projects, such as football reminiscence, disruptive design workshops, exhibitions, and “pop-up” shops. These projects work for people living with dementia, addressing issues around inclusion, participation, creativity and giving an increased sense of self identity through activities that are carefully designed, developed and facilitated in dementia-friendly settings. What you see in this paper are the positive results of co-design projects with people living with dementia, their family members, and their care support workers. These projects have acted as catalysts for conversations and reminiscence on past working lives, personal interests and activities once loved, and they have shone a light on how these opportunities can be exploited to provide amazing benefits for people who are living with dementia. 

Several authors have proposed terms such as “vernacular designers” [32], “silent designers” [33] and “design amateurs” [34] to describe co-design participants such as those described in this paper. Co-design projects have the potential to not only affect the role(s) of the participants in the co-design process, but can also change the role(s) and attitudes of the researcher (designer) [24]. Most of the co-design literature implies a collaborative and cooperative effort between two or more equally able participants. 

This can create significant challenges when working with people living with dementia, however, not all participants will have equal levels of cognitive ability or skills in visual and hands-on activities. This paper, however, describes co-designing with individuals that are not equal in the sense of their cognitive and communication abilities, which brings new challenges to co-design activities and projects. As such, undertaking work of this nature requires careful contemplation and planning. Researchers, therefore, should always ask people living with dementia how they wish to be involved in co-design projects, including at what stages and in what ways they want to be involved. Co-design projects that involve people living with dementia need to include everyone at the outset and ensure that they are involved in setting research priorities. 

Researchers should ask people living with dementia what positive outcomes of the research project might look like for them and must ensure that everyone taking part in the research is physically and emotionally safe at all times. Researchers must use language that is supportive of people with dementia whilst avoiding language that may offend. Researchers also need to be “dementia aware”—they should be compassionate, tolerant, understanding, and respectful whilst working with people living with dementia. Following these guiding principles on how to best conduct co-design projects with people living with dementia will help deliver truly meaningful experiences and outcomes for all involved and ensure that people living with dementia will have full participation and inclusion in all aspects of life, including the right to work.

## Figures and Tables

**Table 1:** Disruptive Design Workshops Details.

	Disrupting Carers	Disrupting Dementia	Disrupting Dementia Tartan	75 BC	Designed with Dementia
Timeframe	2012–2019	2012–2018	2014–2018	2017–2021	2018-today
Aims	To design new forms of care delivery and service design.	To use football to promote reminiscence and intergenerational dementia awareness.	To change attitudes about what people living with dementia can do.	To use the images created by people living with dementia to highlight their inherent creativity.	To showcase the creative potential of people living with dementia.
Methodology Approach	Disruptive, co-design workshops.	Public engagement via national exhibition.	Co-design and making workshops.	Museum visits; Co-design workshops.	Co-design and making workshops.
Participants	c. 50	c. 3000	c. 200	c. 30	c. 100
Partner Organizations	Gateshead Carers; Newcastle Carers	North East Together; Newcastle YMCA; Alzheimer Scotland	Alzheimer Scotland	Alzheimer Scotland	Alzheimer Scotland; Defying Dementia;
Activities	Drawing from memory; sketching everyday objects; 2D/3D modelling; problem-solving challenges.	Manufacture of interactive reminiscence table; engaged with 3000+ museum visitors, football clubs, fans and communities.	National co-design workshops; designing; making; prototyping; national exhibitions of mass-produced tartan.	Visual collage; design and manufacture of a series of textile design prototypes.	Co-design workshops (Lancaster and Glasgow); designing and making products for Pop-Up shop.
Notable Outputs	Funded PhD; redesigned service design.	Interactive football reminiscence table exhibited nationally.	Funded PhD; mass-produced product (tartan) exhibited and sold nationally and globally.	A series of new textile design fabrics based on the life of the Glaswegian comedian Billy Connolly; textiles have been applied to several new products.	Designed with Dementia Pop-Up shop (Lancaster and Glasgow); pop-up shop sells a range of products designed by people living with dementia, such as plates and mugs, tea towels, coasters, tote bags, cushions, make-up bags, ties, etc.

## Data Availability

Not applicable.

## References

[1] Wimo A., Guerchet M., Ali G.C., Wu Y.T., Prina A.M., Winblad B., Jönsson L., Liu Z., Prince M. (2017). The worldwide costs of dementia 2015 and comparisons with 2010. Alzheimer’s Dement..

[2] World Health Organization (2021). Dementia: Key Facts. https://www.who.int/news-room/fact-sheets/detail/dementia.

[3] World Health Organization (2017). Global Action Plan on the Public Health Response to Dementia 2017–2025.

[4] Winton E., Rodgers P.A., Langdon P., Lazar J., Heylighen A., Dong H. (2020). Towards Design and Making Hubs for People Living with Dementia. Designing for Inclusion-Inclusive Design: Looking Towards the Future.

[5] Summers D. (2009). David Cameron Warns of “New Age of Austerity”. The Guardian. https://www.theguardian.com/politics/2009/apr/26/david-cameron-conservative-economic-policy1.

[6] Yeandle S., Buckner L. (2015). Valuing Carers 2015–The Rising Value of Carers’ Support.

[7] Bisson C., Luckner J. (1996). Fun in Learning: The Pedagogical Role of Fun in Adventure Education. Perspectives. J. Exp. Educ..

[8] Ahern J. (2011). From fail-safe to safe-to-fail: Sustainability and resilience in the new urban world. Landsc. Urban Plan..

[9] Christensen C., Overdorf M. (2000). Meeting the Challenge of Disruptive Change. Harv. Bus. Rev..

[10] Scharmer C.O. (2011). Leading from the Emerging Future. Minds for Change–Future of Global Development Ceremony to Mark the 50th Anniversary of the BMZ Federal Ministry for Economic Cooperation and Development.

[11] Rodgers P.A., Tennant A., Dodd K., Salamanca J., Desmet P., Burbano A., Ludden G., Maya J. (2014). Disrupting Health and Social Care by Design. Proceedings of the Colors of Care: The 9th International Conference on Design & Emotion, Bogotá, Colombia, 6–10 October 2014.

[12] Carers U.K. (2019). State of Caring: A Snapshot of Unpaid Care in the UK.

[13] Bury M. (1982). Chronic illness as biographical disruption. Sociol. Health Illn..

[14] Boss P. (1999). Ambiguous Loss: Learning to Live with Unresolved Grief.

[15] Ashworth M., Baker A.H. (2000). Time and Space: Carers’ Views about Respite Care. Health Soc. Care.

[16] Batsch N.L., Mittelman M.S. (2012). World Alzheimer Report 2012: Overcoming the Stigma of Dementia.

[17] Katsuno T. (2005). Dementia from the Inside: How People with Early-stage Dementia Evaluate their Quality of Life. Ageing Soc..

[18] Sanders E.B.N., Stappers P.J. (2014). Probes, Toolkits and Prototypes: Three Approaches to Making in Codesigning. CoDesign.

[19] Kinnaird L. (2012). Delivering Integrated Dementia Care: The 8 Pillars Model of Community Support.

[20] Design Council (2018). The Design Economy 2018: The State of Design in the UK.

[21] Van Klaveren R. Artistic Participatory Practices as a Vehicle for Togetherness. Proceedings of the CUMULUS Conference.

[22] Holcombe S. (2010). The Arrogance of Ethnography: Managing Anthropological Research Knowledge. Aust. Aborig. Stud..

[23] Manzini E., Rizzo F. (2011). Small Projects/Large Changes: Participatory Design as an Open Participated Process. CoDesign.

[24] Winton E., Rodgers P.A., Woodcock A., Moody L., McDonagh D., Jain A., Jain L.C. (2020). Realising the Potential of People Living with Dementia through Co-designing and Making Interventions. Design of Assistive Technology for Ageing Populations.

[25] Schön D. (1982). The Reflective Practitioner: How Professionals Think in Action.

[26] Schon D.A. (1987). Educating the Reflective Practitioner: Toward a New Design for Teaching and Learning in Professions.

[27] Cowan J. (2006). On Becoming an Innovative University Teacher.

[28] Kitwood T. (1998). Toward a Theory of Dementia Care: Ethics and Interaction. J. Clin. Ethics.

[29] Kitwood T. (1997). The Experience of Dementia. Aging Ment. Health.

[30] Kitwood T. (1997). Dementia Reconsidered.

[31] Brooks J., Savitch N., Gridley K. (2017). Removing the ‘Gag’: Involving People with Dementia in Research as Advisers and Participants. Soc. Res. Pract..

[32] Reitan J.B. (2006). Inuit Vernacular Design as a Community of Practice for Learning. CoDesign.

[33] Gorb P., Dumas A. (1987). Silent Design. Des. Stud..

[34] Leadbeater C. (2009). We Think.

